# Severe Hyponatremia-Induced Takotsubo Cardiomyopathy

**DOI:** 10.7759/cureus.88820

**Published:** 2025-07-26

**Authors:** Adnan Ahmed, Ayesha Javaid, David Lai Chin Kon

**Affiliations:** 1 Cardiology, Stockport NHS Foundation Trust, Manchester, GBR; 2 Cardiology, Countess of Chester Hospital, Chester, GBR

**Keywords:** : acute coronary syndrome, apical balloning, cardiomyopathy, hyponatremia, takotsubo

## Abstract

We report the case of a 79-year-old woman with background of chronic hyponatremia secondary to syndrome of inappropriate antidiuretic hormone secretion (SIADH) who presented with chest pain and marked troponin elevation in the absence of an identifiable emotional stressor. Initial investigations revealed severe hyponatremia (serum sodium 122 mmol/L) and elevated cardiac enzymes. Her electrocardiogram showed normal sinus rhythm with deep, symmetrical T wave inversions in the inferior and precordial leads. Other investigations revealed apical akinesia on echocardiography and apical ballooning on the left ventriculogram. Coronary angiography showed unobstructed coronary arteries. Further investigations supported the diagnosis of SIADH, for which she was treated with fluid restriction and demeclocycline. The patient responded well with gradual normalisation of her sodium levels and improvement in symptoms. Follow-up imaging showed normal biventricular systolic function and resolution of apical ballooning. Given the temporal association and lack of alternative triggers, we hypothesize that profound hyponatremia contributed to the development of Takotsubo cardiomyopathy (TCM). This case highlights the potential for severe hyponatraemia to act as a precipitating factor in TCM.

## Introduction

Takotsubo cardiomyopathy (TCM), often termed stress-induced cardiomyopathy, involves a sudden but reversible decrease in left ventricular systolic function, primarily affecting the apex, and is usually provoked by emotional or physical stressors [1]. It is observed in about 1-2% of patients who present with symptoms suggestive of acute coronary syndrome (ACS) [2]. The condition commonly imitates ACS, with similar clinical features such as chest pain, ECG abnormalities, and elevated cardiac biomarkers, but typically occurs with non-obstructed coronary arteries. Although the underlying pathophysiology of TCM is not fully elucidated, proposed mechanisms include myocardial stunning secondary to catecholamine surge, microvascular dysfunction, and endothelial impairment [3]. The literature highlights emotional stressors (e.g., grief or fear), cerebrovascular events, invasive medical procedures, and rare triggers such as endocrine and electrolyte imbalance as the most frequently reported precipitants of TCM [4,5]. Recognition of atypical triggers is particularly important as they may make the diagnosis more challenging.

## Case presentation

A 79-year-old hypertensive woman presented to the emergency department with a two-day history of substernal chest pain radiating to the jaw and left shoulder. She reported no preceding emotional stressors. Her past medical history was significant for syndrome of inappropriate antidiuretic hormone secretion (SIADH), believed to have been induced by prior use of flupentixol, which she had taken previously for the treatment of psychiatric illness. At the time of that diagnosis, she developed seizures secondary to profound hyponatraemia, requiring admission to the intensive care unit. She had remained under endocrine follow-up since, with baseline serum sodium levels ranging from 133 to 140 mmol/L over the preceding year. There was no known history of diabetes mellitus, dyslipidemia, or coronary artery disease. She had a dual-chamber pacemaker implanted four years ago. She was a non-smoker and was taking lencardipine 2.5 mg for hypertension.

On examination, she was alert, oriented, and hemodynamically stable. Blood pressure was 134/78 mmHg, heart rate 86 bpm, respiratory rate 18 breaths/min, and oxygen saturation 98% on room air. Cardiovascular, respiratory, abdominal, and neurological examinations were unremarkable. Initial laboratory investigations at the time of admission (Day 1) demonstrated hyponatraemia, with a serum sodium concentration of 128 mmol/L falling further to 122 mmol/L during admission. Additional investigations are summarised in Table 1.

**Table 1:** Other laboratory investigations. NT-proBNP = N-terminal pro–B-type Natriuretic Peptide; eGFR = Estimated Glomerular Filtration Rate; LDL = Low-Density Lipoprotein, ALT = Alanine Aminotransferase

Test Name	Result	Units	Reference Range
Haemoglobin	130	g/L	115–160 g/L (female)
White Cell Count (WCC)	10.3	x10⁹/L	4.0–11.0 x10⁹/L
Platelet Count	193	x10⁹/L	150–400 x10⁹/L
Sodium	122	mmol/L	135–145 mmol/L
Potassium	3.8	mmol/L	3.5–5.0 mmol/L
NT-proBNP	1475	ng/L	<125 ng/L (<75 years)
Creatinine	62	µmol/L	45–90 µmol/L (female)
eGFR	82	mL/min/1.73 m²	>60 mL/min/1.73 m²
C-reactive protein (CRP)	<4	mg/L	<5 mg/L
Troponin I (initial)	122	ng/L	<16 ng/L (female)
Troponin I (peak)	8098	ng/L	<16 ng/L (female)
Thyroid-Stimulating Hormone (TSH)	3.1	mU/L	0.4–4.0 mU/L
Serum Cortisol	551	nmol/L	140–690 nmol/L (AM)
Normetanephrine	1033	pmol/L	0–1180 pmol/L
Metanephrine	407	pmol/L	0–510 pmol/L
3-Methoxytyramine	<75	pmol/L	0–180 pmol/L
Total Serum Cholesterol	7.0	mmol/L	<5.0 mmol/L
LDL Cholesterol	4.3	mmol/L	<3.0 mmol/L
Triglycerides	1.3	mmol/L	< 1.7 mmol/L
Serum Albumin	42	g/L	34-54 g/L
Total Protein	68	g/L	6-8.3 g/L
ALT	15	U/L	10-40 (Female)
Alkaline Phosphatase	84	U/L	30-130 U/L
Total Bilirubin	12	µmol/L	1.7-20.5 umol/L
Serum glucose	6.5	mmol/L	3.9-5.4 mmol/L
Serum osmolality	274	mmol/kg	275-295 mmol/kg
Urine osmolality	355	mmol/kg	
Urine Sodium	87	mmol/L	

Plasma free metanephrines were within normal limits, effectively ruling out a catecholamine-secreting tumor. Further workup for hyponatremia revealed serum osmolality of 274 mOsm/kg, consistent with hypotonic hyponatremia. Urine analysis demonstrated a urine osmolality of 355 mOsm/kg and a urine sodium concentration of 87 mmol/L. She remained clinically euvolemic. These findings supported a diagnosis of euvolemic hyponatremia, consistent with SIADH.

**Figure 1:**
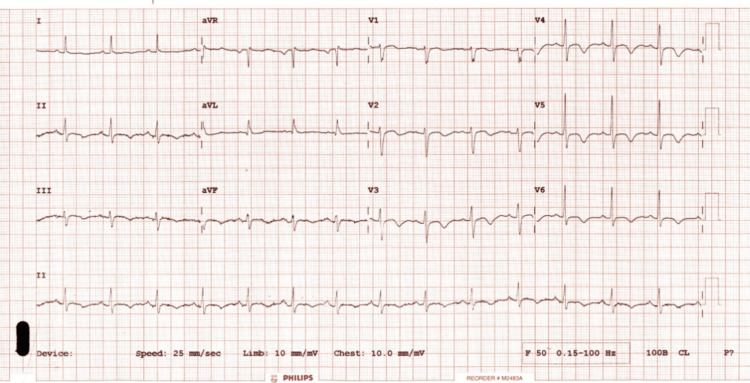
Electrocardiogram showing sinus rhythm and symmetrical T wave inversions in lead II, III, AVF and V2-V6.

The electrocardiogram demonstrated sinus rhythm with symmetrical T wave inversions in the inferior leads (II, III, aVF) and precordial leads (V2 to V6) (Figure 1).

Given the intensity of her chest pain, a CT aortogram was performed to exclude aortic dissection. The imaging demonstrated no evidence of dissection or intramural haematoma, and the thoracic aorta appeared unremarkable. She was started on treatment for acute coronary syndrome.

Transthoracic echocardiography on Day 2 revealed impaired left ventricular systolic function with an estimated ejection fraction of 36-40% based on visual assessment. There were regional wall motion abnormalities consistent with TCM, including akinesia of the mid-apical inferior septum, mid-apical anterolateral wall, apical cap, apical inferior wall, and apical anterior wall. The left ventricular chamber size and wall thickness were within normal limits, and diastolic function was appropriate for the patient's age. The valvular assessment showed trivial aortic regurgitation, mild mitral regurgitation, and mild to moderate tricuspid regurgitation. The estimated pulmonary artery systolic pressure was 40.8 mmHg with an estimated right atrial pressure of 0-5 mmHg. Tricuspid regurgitant velocity (TR Vmax) was measured at 3.2 m/s. She had an intermediate probability of pulmonary hypertension.

**Figure 2:**
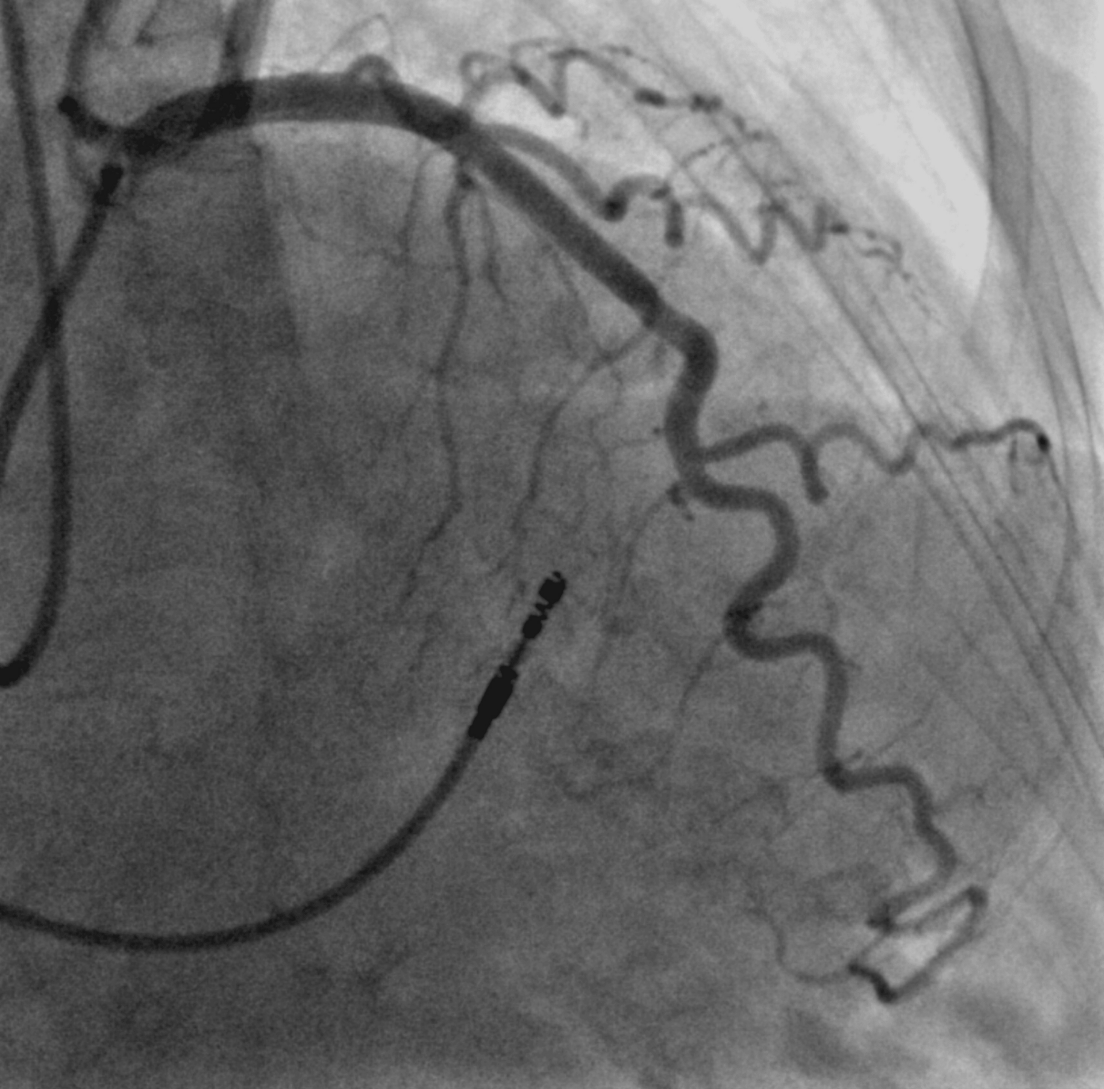
Coronary angiogram revealing unobstructed left anterior descending artery.

**Figure 3:**
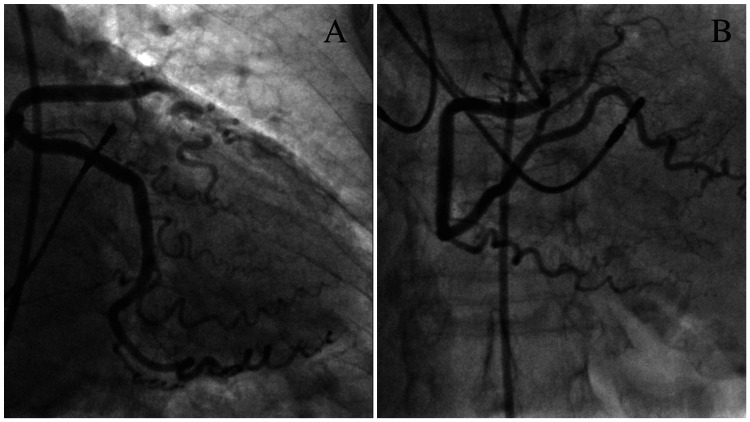
Coronary angiogram showing unobstructed left circumflex artery (A) and the right coronary artery (B)

Invasive coronary angiography on Day 2 demonstrated unobstructed coronary arteries, effectively excluding an ischaemic cause for her presentation (Figure 2, Figure 3A, 3B)

**Figure 4:**
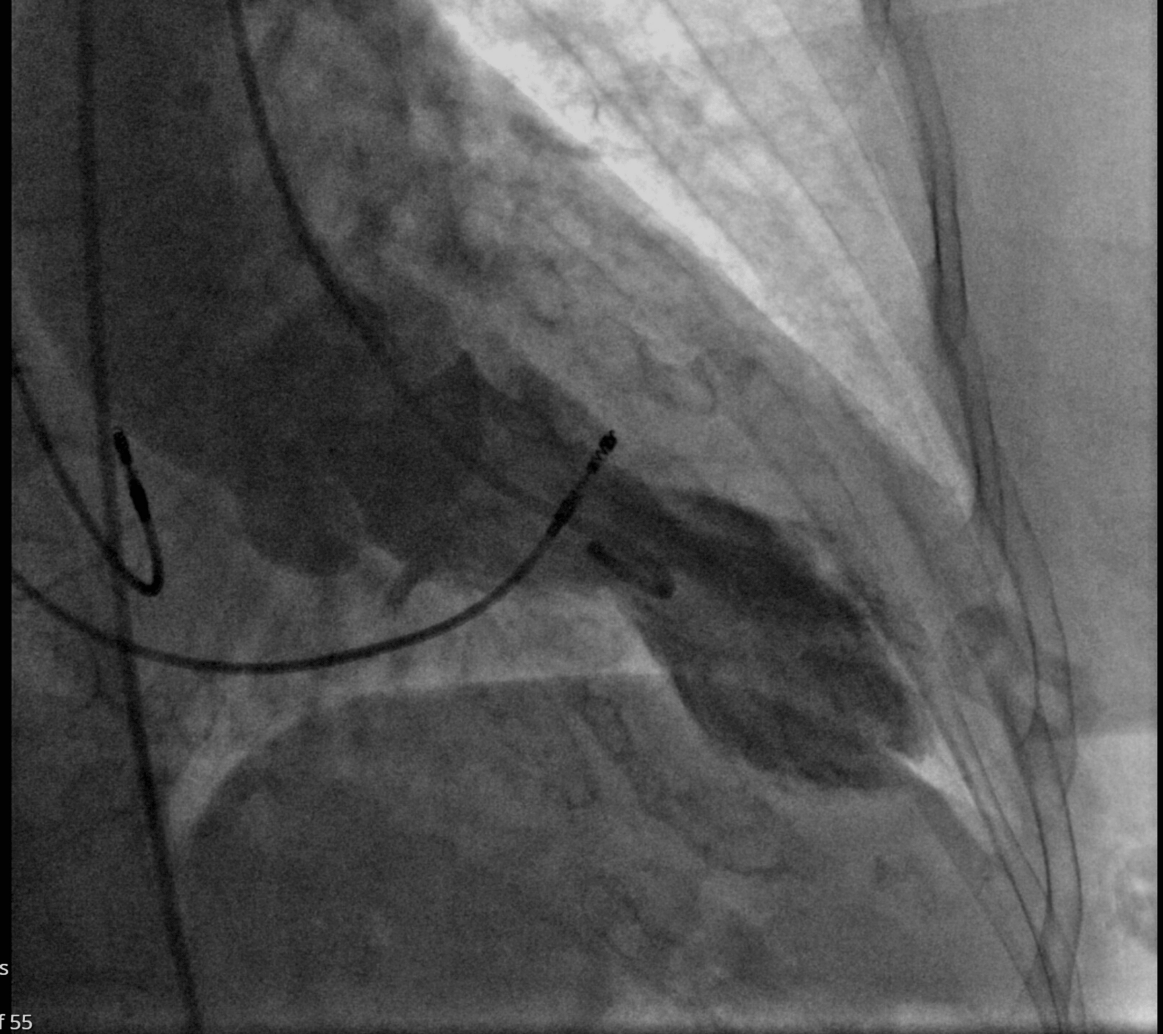
Left ventriculogram demonstrates apical ballooning following left ventricular contrast injection.

Left ventriculography supported the diagnosis of TCM, demonstrating apical ballooning and elevated left ventricular pressures (Figure 4).

The patient was commenced on ramipril 2.5 mg and bisoprolol 2.5 mg and acute coronary treatment was stopped. The endocrinology review identified significant hyponatremia as a possible causative factor, on the background of SIADH. This led to the formulation of a tailored management plan for SIADH, including demeclocycline and fluid restriction. The patient responded well to medical management, and her sodium levels gradually improved over the following two weeks, stabilizing within the range of 133-140 mmol/L. She was discharged on bisoprolol and ramipril. Outpatient cardiac MRI and transthoracic echocardiogram were scheduled for her. Her repeat transthoracic echocardiogram six months later revealed normal left ventricular systolic function with no regional wall motion abnormalities and resolution of apical hypokinesis and ballooning.

## Discussion

TCM, also known as stress-induced cardiomyopathy, is a reversible cardiac disorder that mimics acute coronary syndrome but occurs in the absence of a culprit coronary artery lesion, although it may co-exist with obstructive coronary artery disease [6]. It is often triggered by intense physical or emotional stress and is characterized by transient systolic dysfunction of the left ventricle, commonly involving the apical region, resulting in the classical apical ballooning pattern [7].

Hyponatremia has recently been implicated as both a precipitating factor and a potential consequence of TCM, suggesting a bidirectional relationship [7,8]. In elderly patients, hyponatremia is frequently multifactorial, with contributing factors such as diuretic therapy, hypothyroidism, and renal dysfunction [9]. In our patient, severe hyponatremia was secondary to the SIADH. The link between hyponatremia and TCM has been highlighted in several reports, where acute or profound hyponatremia often due to SIADH, diuretic use, chronic alcohol intake or primary polydipsia was associated with TCM development [1,9-12].

Jha et al. [1] described a 55-year-old woman who developed TCM secondary to profound hyponatremia caused by SIADH following whiplash head injury. Similarly, López-Trejo et al. [9] reported an elderly woman with anginal chest pain and hyponatremia-induced seizures who developed left ventricular apical ballooning confirmed on imaging. Other case reports, including those by Kawano et al. [10] and AbouEzzeddine and Prasad [11], support the association between severe hyponatremia and TCM. Simsek et al. [12] described a rare case of TCM triggered by a combination of acute severe hyponatremia and seizure activity.

The pathophysiology of TCM remains multifactorial, with catecholamine excess considered the primary mechanism [13]. High circulating epinephrine may induce myocardial stunning through a shift from β₂-adrenoceptor Gs-protein to Gi-protein signaling, producing a negative inotropic effect [3,5]. Hyponatremia can exacerbate myocardial susceptibility to stress injury by impairing sodium-calcium exchange, altering cellular excitability, and causing osmotic swelling of cardiac myocytes [5,8]. Acute, rapid declines in sodium are particularly associated with TCM, whereas chronic hyponatremia poses a lower risk [12].

Given the reversible nature of both TCM and hyponatraemia, prompt recognition and correction of the underlying abnormalities are essential to optimize recovery. In the present case, the patient had chronic asymptomatic hyponatremia with stable sodium levels ranging between 133-140 mmol/L over the past year. The acute drop in sodium level, likely due to excessive fluid intake or an unrecognised subclinical intercurrent illness, led her to this presentation. Normalization of serum sodium levels was temporally associated with the resolution of left ventricular dysfunction on follow-up echocardiography, supporting the hypothesis that severe hyponatremia acted as a precipitating factor in the development of TCM. Furthermore, her InterTAK score of 61 strengthened the probability of TCM as the underlying diagnosis.

This case report is limited by its single-patient design. Additional unrecognized factors, including subclinical stress or comorbidities, may have contributed to the clinical presentation. Although myocarditis was deemed unlikely based on normal C-reactive protein levels, definitive exclusion of alternative etiologies requires cardiac magnetic resonance imaging, which remains pending.

## Conclusions

This case illustrates that severe hyponatremia, particularly in the context of SIADH, may act as a precipitating factor for Takotsubo cardiomyopathy in the absence of conventional emotional or physical stressors. The temporal association between worsening hyponatremia and evolving cardiac changes, along with the complete recovery following sodium correction, supports this link. While clinicians should remain vigilant for this possibility especially in elderly patients presenting with myocardial injury without coronary obstruction, the current evidence remains limited and overdiagnosis should be avoided without comprehensive clinical evaluation. Further research is warranted to clarify the pathophysiological mechanisms linking hyponatremia and Takotsubo cardiomyopathy and to identify individuals who may be particularly susceptible to this interaction.
